# Drug-Induced Gingival Overgrowth: A Pilot Study on the Effect of Diphenylhydantoin and Gabapentin on Human Gingival Fibroblasts

**DOI:** 10.3390/ijerph17218229

**Published:** 2020-11-07

**Authors:** Dorina Lauritano, Giulia Moreo, Luisa Limongelli, Elena Tregambi, Annalisa Palmieri, Francesco Carinci

**Affiliations:** 1 Centre of Neuroscience of Milan, Department of Medicine and Surgery, University of Milano-Bicocca, 20126 Milan, Italy; moreo.giulia@gmail.com (G.M.); elena.tregambi@gmail.com (E.T.); 2 Interdisciplinary Department of Medicine, University of Bari, 70121 Bari, Italy; luisa.limongelli@gmail.com; 3 Department of Experimental, Diagnostic and Specialty Medicine, University of Bologna, via Belmoro 8, 40126 Bologna, Italy; plmnls@unife.it; 4 Department of Morphology, Surgery and Experimental Medicine, University of Ferrara, 44121 Ferrara, Italy; crc@unife.it

**Keywords:** diphenylhydantoin, gabapentin, antiepileptic drug (AED), gingival hyperplasia, drug-induced gingival hyperplasia (DIGH)

## Abstract

Introduction. The administration of several classes of drugs can lead to the onset of gingival overgrowth: anticonvulsants, immunosuppressants, and calcium channel blockers. Among the anticonvulsants, the main drug associated with gingival overgrowth is diphenylhydantoin. Materials and Methods. In this study, we compared the effects of diphenylhydantoin and gabapentin on 57 genes belonging to the “Extracellular Matrix and Adhesion Molecule” pathway, present in human fibroblasts of healthy volunteers. Results. Both molecules induce the same gene expression profile in fibroblasts as well as a significant upregulation of genes involved in extracellular matrix deposition like COL4A1, ITGA7, and LAMB3. The two treatments also induced a significant downregulation of genes involved in the expression of extracellular matrix metalloproteases like MMP11, MMP15, MMP16, MMP24, and transmembrane receptor ITGB4. Conclusions. Data recorded in our study confirmed the hypothesis of a direct action of these drugs at the periodontium level, inducing an increase in matrix production, a reduction in its degradation, and consequently resulting in gingival hyperplasia.

## 1. Introduction

Anticonvulsants are drugs used in epilepsy treatment in order to reduce the seizures. To date, the first anticonvulsant choice is diphenylhydantoin (DPH). It is used in 30% of epileptic patients suffering from generalized, complex, partial epilepsy, and cortical focal epilepsy [1,2]. A frequent side effect of this drug, together with immunosuppressant drugs and calcium channel blocking agents, is excessive gingival growth, with a variation ranging from 13% to 50% [3,4]. Drug-induced gingival hyperplasia (DIGH) represents a real concern, since it may cause swelling, bleeding, alteration of chewing, and pronunciation and aesthetics problems, worsening the patient’s quality of life [5]. Kimball was the first of many authors who hypothesized that this drug altered the mechanisms of the host’s immune response, causing an increase in gingival volume or that its chronic use led to a reduction in serum and salivary IgA and to a consequent inflammation of the periodontal tissues [6]. Afterward, the existence of the direct action of anticonvulsant drugs on the periodontium provided by mast cells was supposed: these cells are able to release histamine, heparin, and hyaluronic acid in gingival tissue, which are metabolized by fibroblasts and stimulate collagen production. 

According to the literature, the degree of gingival enlargement in patients receiving anti-convulsing treatment (as well as immunosuppressants and calcium channel blockers) is well correlated with poor plaque control [7]. In fact, the 2014 classification system for periodontal diseases stated that plaque represents a cofactor in the etiology of drug-induced gingival overgrowth [8]. Moreover, the gingival enlargement could make plaque control difficult, leading to a secondary inflammatory process, which aggravates the overgrowth induced by the drug [9]. 

Gabapentin (GB) was introduced in 1994 as an antiepileptic drug (AED) [10], but nowadays is the first drug chosen to treat the postoperative pain. GB is able to reduce the hyperexcitability caused by the presence of lesions in the posterior horn neurons, responsible for central sensitization [11]. 

According to a recent study, the administration of gabapentin may lead to the overexpression of several chemokines and interleukins, causing post-inflammatory gingival enlargement as a side effect [12].

The pathogenesis of DPH and GB induced gingival overgrowth is still unclear: the excessive storage of gingival connective tissue seems to derive from an imbalance between the extracellular matrix (ECM) metabolism (synthesis and degradation) [13]. Some authors suggest that these types of drugs cause a lower degradation of collagen, without an increase in its production [1]. However, other studies have demonstrated that anticonvulsants may enhance type 1 collagen production and alpha-smooth muscle actin expression in human gingival fibroblasts [14]. 

This study aimed at investigating the association between anticonvulsant drugs and the onset of gingival overgrowth, comparing the effects of the diphenylhydantoin molecule with those of gabapentin on the “Extracellular Matrix and Adhesion Molecule” pathway present in human fibroblasts of healthy volunteers. Since GB appears to cause less gingival hyperproliferation with respect to DPH, it was decided to compare the two drugs in order to identify the one that caused fewer side effects.

Anticonvulsant-induced gingival hyperplasia is characterized by fibroblast hyperproliferation in connective tissue with the deposition of extracellular matrix. Cell proliferation also involves epithelial–mesenchymal transition, resulting in changes in the expression of adhesion molecules. For this reason, the secondary aim of this research was to study the molecular mechanisms underlying this modification.

## 2. Materials and Methods

### 2.1. Primary Human Fibroblast Cells Culture

The primary fibroblasts were purchased from ATCC^®^ Cell Lines. Human gingival fibroblasts at the second passage, derived from the tissues of an 11 year old man, 68 year old woman, and 20 year old man, were cultured in DMEM medium (Sigma Aldrich, Inc., St Louis, MO, USA) supplemented with 10% fetal calf serum, antibiotics (penicillin 100 U/mL and streptomycin 100 mg/mL, Sigma Aldrich, Inc., St Louis, MO, USA). Cells were replicated for subsequent experiments. 

Cells were incubated in a humidified atmosphere of 5% CO_2_ at 37 °C. The medium was changed the next day to remove any dead cells that did not adhere to the plate. Subsequently, the changes were made twice a week to permit the cells to produce growth factors that allow them to proliferate. 

### 2.2. Cell Viability Test

A stock solution of DPH 1 mg/mL and GB 1 mg/mL was prepared. 

Fibroblasts were seeded into 96-well plates at a density of 10^4^ cells per well containing 100 µL of cell culture medium and incubated for 24 h to allow cell adherence. Serial dilution of each stock solution was prepared: diphenylhydantoin (5000 ng/mL, 2000 ng/mL, 1000 ng/mL, 500 ng/mL, 100 ng/mL), and gabapentin (5000 ng/mL, 2000 ng/mL, 1000 ng/mL, 500 ng/mL, 100 ng/mL) were prepared.

A set of wells were treated with DPH, three wells for each concentration. Another set of wells were treated with GP. The cell culture medium alone was used as a negative control.

After 24 h of incubation, cell viability was measured using PrestoBlue™ Reagent (Invitrogen, Carlsbad, CA, USA), according to the manufacturer’s instructions [15].

### 2.3. Cell Treatment

Fibroblasts were seeded at a density of 1.0 × 10^5^ cells/mL into 9 cm^2^ (3 mL) wells and incubated for 16 h at 37 °C for the serum starvation

Cells were treated with 1000 ng/mL DPH solution and 1000 ng/mL GP solution for 24 h, prepared in DMEM supplemented with 2% fetal bovine serum (FBS) antibiotics, and amino acids. Cells cultured with medium alone were used as the negative control. 

After the end of the exposure time, RNA was extracted from the cells and processed for the gene expression analyses.Total RNA isolation, cDNA synthesis, and quantitative real-time PCR were performed as described in the previously published article [15].

Custom primers used for amplification, belonging to the “Extracellular Matrix and Adhesion Molecules” pathway, were purchased from Sigma Aldrich (Sigma Aldrich, Inc., St Louis, MO, USA).

### 2.4. Statistical Analysis

Gene expression levels were normalized to the endogenous control gene RPL13 and then calculated as fold changes relative to untreated cells. Quantification was done with the delta/delta Ct calculation method [16].

### 2.5. Detection of Collagen Alpha-4 Levels by Enzyme Linked Immunosorbent Assay

Using sandwich enzyme linked immunoassay (ELISA), the protein levels of Collagen alpha-4 were measured after fibroblast treatment with DPH and GP by using the commercial kit, Human Collagen alpha-4 ELISA Kit (Bioassay Technology Laboratory, Shanghai, China). The protocol for the detection was previously described [15].

COL4A1 levels were expressed as ng COL4A1/ng of total protein.

## 3. Results

DPH and GP solution concentrations to be used for cell treatment were prepared after PrestoBlue™ cell viability test. Based on this test, the concentration that did not significantly affect cell viability for both treatments was 1000 ng/mL.

This concentration was considerably lower than that used in vivo. In vivo, the daily dose of gabapentin is 600–1800 mg. This dose is safe and significantly reduces the frequency of convulsive crisis [17]. The usual dose of phenytoin to treat epilepsy in adults is 300 mg day [18].

The results obtained in vitro are indicative of a possible mechanism of action of anticonvulsant drugs on cells. However, these models need further study concerning both the treatment timing, concentration, and the choice of models that can mimic in vivo conditions as much as possible.

Table 1 and Table 2 report the list gene and their fold change after treatment with DPH and GP respectively, analyzed using Real Time PCR. Bold fonts indicate significant variation of thee gene expression level of the 57 genes belonging to the “Extracellular Matrix and Adhesion Molecules” pathway. A fold change ≥ 2 and *p* value ≤ 0.05 for upregulated genes, and fold change ≤ 0.5 and *p* value ≤ 0.05 for significantly downregulated genes. 

Table 3 and Table 4 report gene expression levels after 24 h treatment with DPH and GB, respectively, compared with the untreated cells.

The two treatments induced the same gene expression profiling in fibroblasts after 24 h of incubation.

Both treatments induce a significant upregulation of genes involved in extracellular matrix deposition like COL4A1, ITGA7, and LAMB3.

The COL4A1 levels measured by enzyme linked immunoassay (ELISA) after diphenylhydantoin and gabapentin treatments showed an expected increase in COL4A1 levels (1.87-fold ± 0.3 for diphenylhydantoin treatment and 1.59-fold ± 0.2 for gabapentin treatment) in treated fibroblasts vs. untreated control, confirming the gene expression results obtained in Real Time PCR. 

Among the significant downregulated genes induced by the two treatments, most were extracellular matrix metalloproteases MMP11, MMP15, MMP16, and MMP24. Other genes significantly downregulated following both treatments were transmembrane receptor ITGB4, and the basement membrane constituent LAMA2, LAMB1, and LAMB3. Other downregulated genes in treated cells were COL7A1 and FN1.

**Figure 1:**
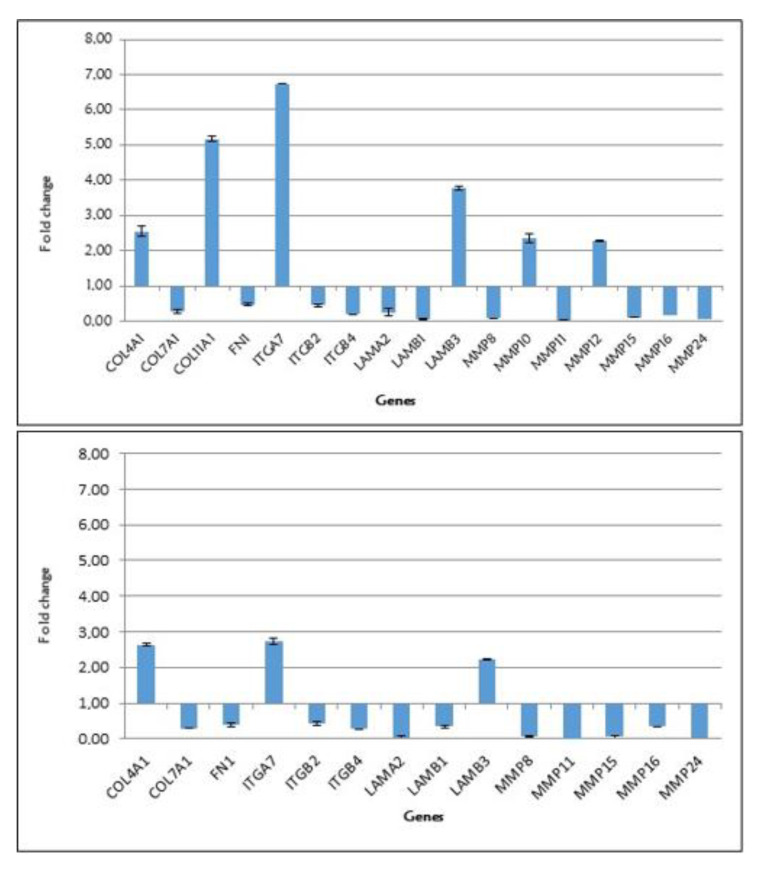
Significant expression levels of the genes up- and downregulated in fibroblast cells treated with diphenylhydantoin (chart above) and gabapentin (chart below).

Figure 1 shows the significant expression levels of the genes up- and downregulated in fibroblast cells treated with diphenylhydantoin (a) and gabapentin (b).

## 4. Discussion

Gingival enlargement is characterized by an excessive growth of periodontal tissue [19] and is the result of an increase in extracellular tissue volume. This pathology is characterized by clinical symptoms like pain, bleeding, abnormal tooth movement, periodontal disorders as well as aesthetic changes, but also occlusion problems, increase of caries development, and periodontal diseases. There are severe forms in which the clinical crown of the dental elements is almost completely covered by gingival tissue. The degree of inflammation, fibrosis, and cellularity depends on the duration and dose of the drug and on the oral hygiene level of the individual [20,21,22,23]. Gingival overgrowth generally appears within three months after the start of the drug administration: it initially impacts the region of interdental papillae, usually at the level of mandibular anteriors, and then extends to adjacent papillae [24]. In the article by Csiszar et al. [25], the expression of molecules normally induced in wound healing (αvβ6 integrin, fibronectin-EDB and -EDA, tenascin-C, type I procollagen, TGF-β, CTGF, and SOS-1) was recorded to be higher in the interdental papilla than in marginal gingival, suggesting that interdental papilla has distinct cellular and molecular properties from other parts of gingiva: specific interdental papilla cell activated phenotypes may be responsible for the predisposition of tissue to gingival enlargement when exposed to additional activating factors such as drug administration. 

Our study focused on the effect of diphenylhydantoin and gabapentin on gingival tissues, investigating the relationship between these two drugs and the onset of gingival overgrowth.

DPH is administered orally and presents a binding capacity with plasma protein higher than 90%. After the oral administration, GP is absorbed at the intestinal level and, unlike DPH, it does not bind plasma proteins. The therapeutic range of DPH lies in most cases between 10 and 20 mL/L and its plasma peak occurs 4–8 h after the ingestion of a single dose. DPH is metabolized by the liver and excreted by the kidney, while GP is excreted unchanged in urine. 

During our experiment, we tested only the most appropriate drug concentrations to produce an effect in gene expression without causing cell death (1000 ng/mL for both treatments after the cell viability test). 

Hassel et al. [26] proved that cells treated with DPH showed a higher level of protein synthetic activity than cells derived from non-treated individuals and that 20% of the protein synthesized by the treated cells was collagen. Kato et al. [1] studied the effect of phenytoin upon collagen degradation and demonstrated that this drug caused impaired collagen degradation through MMPs/TIMP-1 imbalance (metallopeptidase inhibitor-1), leading to collagen accumulation and resulting in gingival enlargement. 

In the literature, it has been demonstrated that age, genetic predisposition, gingival inflammation, and the presence of preexisting plaque may represent risk factors for gingival overgrowth [27].

The first studies in the literature reported that DPH alkalinity could play an important role in the onset of this side effect [28,29,30]. The study by Candotto et al. describes the effect of the gabapentin molecule on 29 genes belonging to the “Inflammatory Cytokines and Receptors” pathway present in human fibroblasts: only one gene (CCL1) resulted up-expressed [1]. A similar study was conducted for the diphenylhydantoin molecule and the result was analogue: among the 29 genes investigated, only 13 genes were statistically significant, and the BMP2 gene was the only one that showed up-expression [31], probably because the studies were performed on healthy people. In our study, both drug molecules induced in treated cells, compared to those untreated, a statistically significant up-regulation of gene involved in extracellular matrix deposition (COL4A1, ITGA7, LAMB3) and a downregulation of extracellular matrix metalloproteases (MMP11, MMP15, MMP16, MMP24), which operate in the extracellular environment of cells and degrade both matrix and non-matrix proteins. This would confirm the hypothesis of a direct action of the drugs at the periodontium level [6]. In particular, the effects of diphenylhydantoin and gabapentin are the alteration of extracellular matrix metabolism; fibroblasts are induced to increase the matrix production and to decrease its degradation. The data suggested that both diphenylhydatoin and gabapentin may lead to the onset of gingival enlargement by contributing to extracellular matrix deposition of human gingival fibroblasts. The role of matrix metalloproteinases in oral diseases was well analyzed in the study by Maciejczyk et al. [32]: MMPs regulate the catabolic turnover of extracellular matrix components (ECM) and also several non-ECM bioactive substrates such as growth factors, cytokines, chemokines, and cell receptors. The lack of balance between the concentration of MMPs and their inhibitors (tissue inhibitors of metalloproteinases, TIMPs) may cause pathological changes including tissue remodeling, inflammation, and uncontrolled ECM turnover. 

Comparing the effect of DPH with other drugs that can lead to gingival enlargement as a side effect, the immunosuppressive drug cyclosporine A (CsA), as another study [33] reported, can induce gingival fibroblasts to inhibit the secretion of several matrix metalloproteases (MMP8, MMP11, MMP15, MMP16, MMP24, and MMP26) and may lead to a downregulation of two integrins (ITGβ2 and ITGβ4), contributing to the accumulation of ECM in the gingival connective tissue and reducing collagen phagocytosis.

With regard to transforming growth factors beta, no significant expression changes were recorded in our experiment and this result supports the hypothesis for which DPH and GB cause a direct regulation of ECM and gingival fibroblasts proliferation. This thesis is in contrast with the data reported in the study by Trackman et al. [34], which suggested that the deregulated balances of cytokines may represent the primary mechanism for the onset of drug-induced gingival enlargement: abnormally high levels of specific cytokines were found in drug induced gingival overgrowth tissues, indicating that certain substances (including DPH) lead to gingival enlargement by altering the normal balance of gingival cytokines. 

The meta-analysis by Wang et al. [35] analyzed the association between transforming growth factor beta-1 (TGF-ß1) gene polymorphism and CsA induced gingival overgrowth; the authors suggested that the codon 10 polymorphism in TGF-ß1 is not associated with susceptibility to CsA-induced gingival enlargement. However, while it has been established that TGF-ß1 plays a crucial role in the pathogenesis of CsA-induced gingival enlargement, the data reported in the literature regarding this point are controversial. Some studies are in contrast with those by Wang et al.: Dunning et al. [36] confirmed the hypothesis reported by previous studies [37] that demonstrated that the proline form of TGF-ß1 secretion was higher than that of the leucine form of TGF-ß1 at codon 10, causing the increase in protein production. 

Although the literature reported a higher expression of fibronectin-1 gene associated with phenytoin administration, data obtained in our study showed a downregulation of this gene after the treatment with both GP and DPH. According to Sume et al. [38], phenytoin-induced overgrowth tissues present higher levels of fibronectin in connective tissue fibroblasts in both subsulcular and suboral regions underlying the epithelium. The authors suggested that the epithelial to mesenchymal transition (EMT) process may contribute to human gingival enlargement and fibrosis. EMT consists of weakening the epithelial cell–cell and cell–extracellular matrix interaction and differentiation of epithelial cells into fibrogenic fibroblast-like cells: the increase in the expression of fibroblast markers including fibronectin is a typical event in this process.

The study by Myrillas et al. [39] demonstrated that gingival enlargement induced by immunosuppressants (CsA) is characterized by higher levels of interleukin-6 (IL-6) and interleukin-1ß (IL-1ß). The alteration in the concentrations of these two cytokines may play a central role in the pathogenesis of drug induced gingival overgrowth: IL-1ß leads to the production of metalloproteinase (MMP), inducing connective tissue degradation, while IL-6 is responsible for the increase of metallopeptidase expression (TIMP), inhibiting tissue breakdown. 

Anticonvulsant monotherapy has been considered the ideal treatment of epilepsy, but some articles have demonstrated that in around 30% of patients, epilepsy did not fully respond to anticonvulsant monotherapy. In that case, polypharmacy with anticonvulsants is the only option [40]: in the future, it would be interesting to investigate whether polypharmacy with anticonvulsants may enhance the risk of drug-induced gingival enlargement. Further research should be performed in order to clarify the entity of the role of gingival fibroblasts in the development of drug-induced gingival overgrowth and to what extent other mechanisms (altered cytokines regulation) may be responsible for this side effect. 

In conclusion, the recent article by Assaggar et al. demonstrated the ability of lovastatin, an approved drug used especially for the treatment of hypercholesteremia, to prevent the development of phenytoin-induced gingival overgrowth in mouse model: lovastatin seemed to attenuate epithelial gingival tissue growth in phenytoin-treated mice and altered the expressions of markers for epithelial to mesenchymal transition [41].

## 5. Strengths and Limitations of the Study

Human gingival fibroblasts were cultured and treated following a rigorous methodology. The same markers were analyzed for both the DPH treated cells and GB treated one, allowing for the comparison between these two cell cultures. Our data were obtained from an in vitro experiment, but further studies should be conducted, in which treatment timing, concentration, and type of chosen models mimic the in vivo conditions as much as possible. The literature provides limited studies regarding the effect of GP on gingival tissues and, for this reason, some of the references included in our article are not recent.

## 6. Conclusions

This paper treated human gingival fibroblasts with diphenylhydantoin and gabapentin in order to analyze the expression profile of 57 genes belonging to the “Extracellular Matrix and Adhesion Molecules”. Data recorded in this study may confirm that anticonvulsant drug administration may cause the increase in matrix deposition and, at the same time, the reduction of its degradation, leading to the onset of gingival overgrowth: thus, extracellular matrix deposition genes (COL4A1, ITGA7, LAMB3) of the treated cells showed an upregulation, while extracellular metalloproteases (MMP11, MMP15, MMP16, and MMP24) were significantly inhibited. 

## Figures and Tables

**Table 1:** Gene expression profile of 57 genes belonging to the “Extracellular Matrix and Adhesion Molecules” pathway analyzed using Real time PCR after 24 h of treatment with **diphenylhydantoin.**

Gene	Fold Change	Gene Function
CD44	0.88	Cell–Cell Adhesion
CDH1	1.54	Cell–Cell Adhesion
COL1A2	1.22	Collagens & Extracellular Matrix Structural constituent
COL2A1	0.88	Collagens & Extracellular Matrix Structural constituent
COL3A1	0.67	Collagens & Extracellular Matrix Structural constituent
COL4A1	**2.54**	Collagens & Extracellular Matrix Structural constituent
COL5A1	1.11	Collagens & Extracellular Matrix Structural constituent
COL6A1	1.32	Collagens & Extracellular Matrix Structural constituent
COL7A1	**0.27**	Collagens & Extracellular Matrix Structural constituent
COL8A1	1.35	Collagens & Extracellular Matrix Structural constituent
COL9A1	0.89	Collagens & Extracellular Matrix Structural constituent
COL10A1	1.00	Collagens & Extracellular Matrix Structural constituent
COL11A1	**5.17**	Collagens & Extracellular Matrix Structural constituent
CCTNA1	1.84	Cell Adhesion Molecule
CTNB	1.36	Cell Adhesion Molecule
CTNND2	0.77	Cell Adhesion Molecule
FN1	**0.47**	Cell Adhesion Molecule
HAS1	1.05	Transmembrane Receptor
ILF3	0.90	Transmembrane Receptor
ITGA1	1.58	Transmembrane Receptor
ITGA2	1.92	Transmembrane Receptor
ITGA3	1.60	Transmembrane Receptor
ITGA4	1.54	Transmembrane Receptor
ITGA5	1.79	Transmembrane Receptor
ITGA6	1.09	Transmembrane Receptor
ITGA7	**6.73**	Transmembrane Receptor
ITGA8	1.73	Transmembrane Receptor
ITGB1	1.59	Transmembrane Receptor
ITGB2	**0.44**	Transmembrane Receptor
ITGB4	**0.19**	Transmembrane Receptor
ITGB5	1.14	Transmembrane Receptor
LAMA1	0.77	Basement Membrane Constituent
LAMA2	**0.26**	Basement Membrane Constituent
LAMA3	1.05	Basement Membrane Constituent
LAMB1	**0.06**	Basement Membrane Constituent
LAMB2	1.50	Basement Membrane Constituent
LAMB3	**3.78**	Basement Membrane Constituent
MMP2	1.43	Extracellular Matrix Protease
MMP3	1.15	Extracellular Matrix Protease
MMP7	1.55	Extracellular Matrix Protease
MMP8	**0.08**	Extracellular Matrix Protease
MMP9	0.80	Extracellular Matrix Protease
MMP10	**2.36**	Extracellular Matrix Protease
MMP11	**0.02**	Extracellular Matrix Protease
MMP12	**2.29**	Extracellular Matrix Protease
MMP13	1.48	Extracellular Matrix Protease
MMP14	0.79	Extracellular Matrix Protease
MMP15	**0.10**	Extracellular Matrix Protease
MMP16	**0.18**	Extracellular Matrix Protease
MMP24	**0.05**	Extracellular Matrix Protease
MMP26	1.74	Extracellular Matrix Protease
TGFB1	1.12	TGFβ Signaling
TGFB2	0.94	TGFβ Signaling
TGFB3	0.73	TGFβ Signaling
TIMP1	0.88	Extracellular Matrix Protease Inhibitor
VCAN	0.86	Cell Adhesion Molecule
RPL13	1.00	Housekeeping gene

**Table 2:** Gene expression profile of 57 genes belonging to the “Extracellular Matrix and Adhesion Molecules” pathway analyzed using Real time PCR after 24 h of treatment with **gabapentin.**

Gene	Fold Change	Gene Function
CD44	0.64	Cell–Cell Adhesion
CDH1	1.71	Cell–Cell Adhesion
COL1A2	0.96	Collagens & Extracellular Matrix Structural constituent
COL2A1	0.88	Collagens & Extracellular Matrix Structural constituent
COL3A1	0.92	Collagens & Extracellular Matrix Structural constituent
COL4A1	**2.64**	Collagens & Extracellular Matrix Structural constituent
COL5A1	0.73	Collagens & Extracellular Matrix Structural constituent
COL6A1	0.82	Collagens & Extracellular Matrix Structural constituent
COL7A1	**0.30**	Collagens & Extracellular Matrix Structural constituent
COL8A1	0.98	Collagens & Extracellular Matrix Structural constituent
COL9A1	0.78	Collagens & Extracellular Matrix Structural constituent
COL10A1	0.87	Collagens & Extracellular Matrix Structural constituent
COL11A1	1.36	Collagens & Extracellular Matrix Structural constituent
CCTNA1	1.12	Cell Adhesion Molecule
CTNB	0.99	Cell Adhesion Molecule
CTNND2	0.73	Cell Adhesion Molecule
FN1	**0.39**	Cell Adhesion Molecule
HAS1	0.79	Transmembrane Receptor
ILF3	0.78	Transmembrane Receptor
ITGA1	1.41	Transmembrane Receptor
ITGA2	1.64	Transmembrane Receptor
ITGA3	1.27	Transmembrane Receptor
ITGA4	0.89	Transmembrane Receptor
ITGA5	1.22	Transmembrane Receptor
ITGA6	0.74	Transmembrane Receptor
ITGA7	**2.73**	Transmembrane Receptor
ITGA8	0.62	Transmembrane Receptor
ITGB1	1.30	Transmembrane Receptor
ITGB2	**0.43**	Transmembrane Receptor
ITGB4	**0.27**	Transmembrane Receptor
ITGB5	0.76	Transmembrane Receptor
LAMA1	0.64	Basement Membrane Constituent
LAMA2	**0.05**	Basement Membrane Constituent
LAMA3	0.65	Basement Membrane Constituent
LAMB1	**0.35**	Basement Membrane Constituent
LAMB2	0.91	Basement Membrane Constituent
LAMB3	**2.23**	Basement Membrane Constituent
MMP2	0.97	Extracellular Matrix Protease
MMP3	1.17	Extracellular Matrix Protease
MMP7	1.41	Extracellular Matrix Protease
MMP8	**0.07**	Extracellular Matrix Protease
MMP9	0.80	Extracellular Matrix Protease
MMP10	1.34	Extracellular Matrix Protease
MMP11	**0.004**	Extracellular Matrix Protease
MMP12	1.57	Extracellular Matrix Protease
MMP13	1.36	Extracellular Matrix Protease
MMP14	1.19	Extracellular Matrix Protease
MMP15	**0.05**	Extracellular Matrix Protease
MMP16	**0.34**	Extracellular Matrix Protease
MMP24	**0.05**	Extracellular Matrix Protease
MMP26	1.08	Extracellular Matrix Protease
TGFB1	1.19	TGFβ Signaling
TGFB2	0.88	TGFβ Signaling
TGFB3	0.85	TGFβ Signaling
TIMP1	0.87	Extracellular Matrix Protease Inhibitor
VCAN	0.82	Cell Adhesion Molecule
RPL13	1.00	Housekeeping gene

**Table 3:** Significant gene expression levels after 24 h of treatment with **diphenylhydantoin** compared with the untreated cells.

Gene	Fold Change	SD (+/–)	Gene Function
COL4A1	2.54	0.07	Collagens & Extracellular Matrix Structural constituent
COL7A1	0.27	0.01	Collagens & Extracellular Matrix Structural constituent
COL11A1	5.17	0.15	Collagens & Extracellular Matrix Structural constituent
FN1	0.47	0.05	Cell Adhesion Molecule
ITGA7	6.73	0.08	Transmembrane Receptor
ITGB2	0.44	0.05	Transmembrane Receptor
ITGB4	0.19	0.00	Transmembrane Receptor
LAMA2	0.26	0.03	Basement Membrane Constituent
LAMB1	0.06	0.00	Basement Membrane Constituent
LAMB3	3.78	0.09	Basement Membrane Constituent
MMP8	0.08	0.00	Extracellular Matrix Protease
MMP10	2.36	0.05	Extracellular Matrix Protease
MMP11	0.02	0.00	Extracellular Matrix Protease
MMP12	2.29	0.13	Extracellular Matrix Protease
MMP15	0.10	0.00	Extracellular Matrix Protease
MMP16	0.18	0.02	Extracellular Matrix Protease
MMP24	0.05	0.00	Extracellular Matrix Protease

**Table 4:** Significant gene expression levels after 24 h of treatment with **gabapentin** compared with the untreated cells.

Gene	Fold Change	SD (+/–)	Gene Function
COL4A1	2.64	0.04	Collagens & Extracellular Matrix Structural constituent
COL7A1	0.30	0.04	Collagens & Extracellular Matrix Structural constituent
FN1	0.39	0.01	Cell Adhesion Molecule
ITGA7	2.73	0.06	Transmembrane Receptor
ITGB2	0.43	0.08	Transmembrane Receptor
ITGB4	0.27	0.04	Transmembrane Receptor
LAMA2	0.05	0.00	Basement Membrane Constituent
LAMB1	0.35	0.03	Basement Membrane Constituent
LAMB3	2.23	0.03	Basement Membrane Constituent
MMP8	0.07	0.00	Extracellular Matrix Protease
MMP11	0.004	0.00	Extracellular Matrix Protease
MMP15	0.05	0.00	Extracellular Matrix Protease
MMP16	0.34	0.04	Extracellular Matrix Protease
MMP24	0.05	0.00	Extracellular Matrix Protease

## Abbreviations

**AED** = antiepileptic drug; **CCL1** = C-C motif chemokine ligand 1; **CDH1** = cadherin 1; **CD44** = CD44 molecule (Indian blood group); **COL1A2** = collagen type I alpha 2 chain; **COL2A1/ COL3A1/ COL4A1/ COL5A1/ COL6A1/ COL7A1/ COL8A1/ COL9A1/ COL10A1/ COL11A1** = collagen type II/III/IV/V/VI/VII/VIII/IX/X/XI alpha 1 chain; **CTNB** = countin-2 precursor; **CTGF** = connective tissue growth factor; **CTNNA1** = catenin alpha 1; **CTNNB1** = catenin beta 1; **CTNND2** = catenin delta 2; **DIGH** = drug-induced gingival hyperplasia; **DMEM** = Dulbecco’s modified Eagle medium; **DPH** = Diphenylhydantoin; **ECM** = extracellular matrix; **ELISA** = sandwich enzyme linked immunoassay; **GB** = Gabapentin; **FN1** = fibronectin-1; **HAS1** = Hyaluronan Synthase 1; **ILF3** = Interleukin enhancer-binding factor 3; **ITGA1/ ITGA2/ ITGA3/ ITGA4/ ITGA5/ ITGA6/ ITGA7/ ITGA8** = integrin subunit alpha 1/2/3/4/5/6/7/8; **ITGB1/ ITGB2/ ITGB3/ ITGB4/ ITGB5** = integrin subunit beta 1/2/3/4/5; **LAMA 1/ LAMA2/ LAMA3** = laminin subunit aplha 1/2/3; **LAMB1/ LAMB2/ LAMB3** = laminin subunit beta 1/2/3; **MMP2/ MMP3/ MMP7/ MMP8/ MMP9/ MMP10/ MMP11/ MMP12/ MMP13/ MMP14/ MMP15/ MMP16/ MMP24/ MMP26** = matrix metallopeptidase 2/3/7/8/9/10/11/12/13/14/15/16/24/26; **PCR** = polymerase chain reaction; **SOS-1** = RasRac guanine nucleotide Exchange factor1; **TGFB1/ TGFB2/ TGFB3** = transforming growth factor beta 1/2/3; **TIMP1** = metallopeptidase inhibitor 1; **VCAN** = versican; **RPL13** = ribosomal protein L13.
